# Breviscapine Injection Improves the Therapeutic Effect of Western Medicine on Angina Pectoris Patients

**DOI:** 10.1371/journal.pone.0129969

**Published:** 2015-06-08

**Authors:** Chuan Wang, Yafeng Li, Shoucui Gao, Daxin Cheng, Sihai Zhao, Enqi Liu

**Affiliations:** 1 Research Institute of Atherosclerotic Disease, Xi’an Jiaotong University Cardiovascular Research Center, Shaanxi, China; 2 Department of Molecular Pathology, Interdisciplinary Graduate School of Medicine and Engineering, University of Yamanashi, Yamanashi, Japan; 3 The Third Affiliated Hospital of the School of Medicine, Xi’an Jiaotong University, Shaanxi, China; Kurume University School of Medicine, JAPAN

## Abstract

To evaluate the beneficial and adverse effects of breviscapine injection in combination with Western medicine on the treatment of patients with angina pectoris. The Cochrane Central Register of Controlled Trials, Medline, Science Citation Index, EMBASE, the China National Knowledge Infrastructure, the Wanfang Database, the Chongqing VIP Information Database and the China Biomedical Database were searched to identify randomized clinical trials (RCTs) that evaluated the effects of Western medicine compared to breviscapine injection plus Western medicine on angina pectoris patients. The included studies were analyzed using RevMan 5.1.0 software. The literature search yielded 460 studies, wherein 16 studies matched the selection criteria. The results showed that combined therapy using Breviscapine plus Western medicine was superior to Western medicine alone for improving angina pectoris symptoms (OR =3.77, 95% Cl: 2.76~5.15) and also resulted in increased electrocardiogram (ECG) improvement (OR=2.77, 95% Cl: 2.16~3.53). The current evidence suggests that Breviscapine plus Western medicine achieved a superior therapeutic effect compared to Western medicine alone.

## Introduction

Cardiovascular and cerebrovascular diseases are the leading cause of death in China and Western countries; more than 40% of people die of these diseases [1]. Coronary heart disease (CHD) is one of the main threats to health both in developed and developing countries. Angina pectoris due to CHD is a common clinical syndrome that is caused by temporary and rapid myocardial ischemia and anoxia [2]. According to the latest data from the World Health Organization in 2011, China has the second highest number of deaths due to CHD in the world [3]. Managing angina pectoris well is one of the keys to reducing the mortality rate of CHD [4]. Angina pectoris has been identified as a predictive factor for an increased risk of heart attack, cardiac arrest, and sudden cardiac death [4]. A prospective cohort study showed that 65% of patients with angina pectoris frequently suffer from recurrent attacks after the first attack of angina,15% progress to non- fatal myocardial infarction, and 15% of the patients with abnormal electrocardiograph (ECG) findings died within the seven-year follow-up period [5]. Therefore, controlling the frequency of angina pectoris attacks, improving the patient's ECG abnormalities, and the prevention of myocardial infarction, as well as reducing the mortality rate are important goals for the treatment of angina pectoris. Currently, routine clinical treatments for angina pectoris mainly consist of nitroglycerin, β-receptor blocking drugs, calcium channel antagonists, nitrates, and oxygen therapy [6]. However, these drugs also exhibit different degrees of adverse effects. For example, rapid withdrawal from β-receptor blocking drugs may induce the rapid onset of acute coronary events [7]. Chinese herbal medicine has occupied an important position in the health system of China. It has a thousand of years of history and has been widely used in clinical practice. An increasing number of international clinicians have also shown an interest in the clinical studies of Chinese herbal medicine. Dengzhanhua is the dried whole plant of *Erigeron breviscapus* (Vant) Hand-Mazz, also known as fleabane and Dong-ju, which is mainly found in southwest China, especially in Yunnan [8]. Breviscapine is the total flavonoid component extracted from *Erigeron breviscapus* (Vant) Hand-Mazz. Breviscapine can dilate capillaries, reduce vascular resistance and platelet aggregation, produce anti-coagulation effects, scavenge free radicals, and improve microcirculation [9–13]. Breviscapine, in conjunction with Western Medicine, has been widely used for the treatment of CHD in China. However, the current clinical data evaluating the therapeutic effect of breviscapine on angina pectoris patients is limited due to the lack of large-sample randomized controlled trials. Therefore, it is necessary to perform a meta-analysis to combine and assess small-sample randomized clinical trials (RCTs) for the efficacy of breviscapine plus Western medicine compared to Western medicine alone.

## Materials and Methods

### Literature searches

Worldwide databases including the Cochrane Central Register of Controlled Trials, Medline, Science Citation Index, EMBASE and Chinese databases such as the China National Knowledge Infrastructure (CNKI), WanFang Database (WF), Chinese BioMedical Literature Database (CBM) and Chongqing VIP Information Database (CQVIP) were searched to identify randomized controlled trials that reported the effects of Western medicine with or without Breviscapine injection (Dengzhanhuasu Injection) therapies in angina pectoris patients. The last retrieval in all databases was performed on March 12, 2013. The keywords used in the literature searches included the following: angina pectoris, Breviscapine, Dengzhanhuasu, Chinese traditional medicine, Chinese traditional drugs, herbs and trial.

### Selection Criteria

The eligibility criteria included the following: (1) the design of randomized controlled trials was explicitly described; (2) participants were suffering from and being treated for angina pectoris; (3) Breviscapine injection was used only in the treatment group. Western medicine therapies included the use of nitroglycerin, beta blockers, calcium channel blockers, nitrate esters or oxygen inhalation; and (4) patients were treated for at least 2 weeks. The exclusion criteria were as follows: (1) duplication of a previous study, reports of duplicated studies were excluded by examining the author list, parent institution, sample size and results; (2) the diagnostic criteria and treatment criteria for angina pectoris were not specified; and (3) the study did not include symptomatic and ECG improvement as the major outcome.

### Criteria for Symptomatic and ECG Improvements

“Effective” was defined as the disappearance of angina pectoris symptoms or a noticeable improvement. At least a 50% reduction in symptoms was defined as “improvement”, while more than an 80% reduction was defined as “marked improvement”. Significant ECG improvements should achieve at least a 0.05 mV elevation of the ST segment in the ECG (improved) or nearly normal (markedly improved) ECG findings during an exercise test.

### Study Selection and data collection

All potential studies were independently screened by two authors (Chuan Wang and Yafeng Li) according to the eligibility criteria, and any disagreements were subsequently resolved by discussion. The quantitative data included publication year; name of the authors; methods; sample size; dosage and treatment duration; outcome measures and adverse events.

### Assessment of study quality

The methodological quality was assessed by two authors (Daxin Cheng and Shoucui Gao) based on the Cochrane quality evaluation standard. High quality trials should obtain a high Jadad Score and clearly describe the adequate randomization methods, allocation concealment, blinding method, withdrawal reasons and management of incomplete outcome data [14–15]. Extracted data were transferred to Review Manager 5.1 (Cochrane IMS, Copenhagen, Denmark) for the meta-analysis.

### Statistical Analysis

The software Review Manager (RevMan) 5.1 was used for the meta-analysis. The odds ratio (OR) with a 95% confidence interval (CI) was used as the main measure of association. Heterogeneity was explored using a forest plot constructed by RevMan software. Due to the lower anticipated variability among trials, the OR value in the fixed-effects model was calculated, and when the 95% CI did not include the value of 1.0, the factor was considered statistically significant.

## Results

### Study Selection

**Fig 1:**
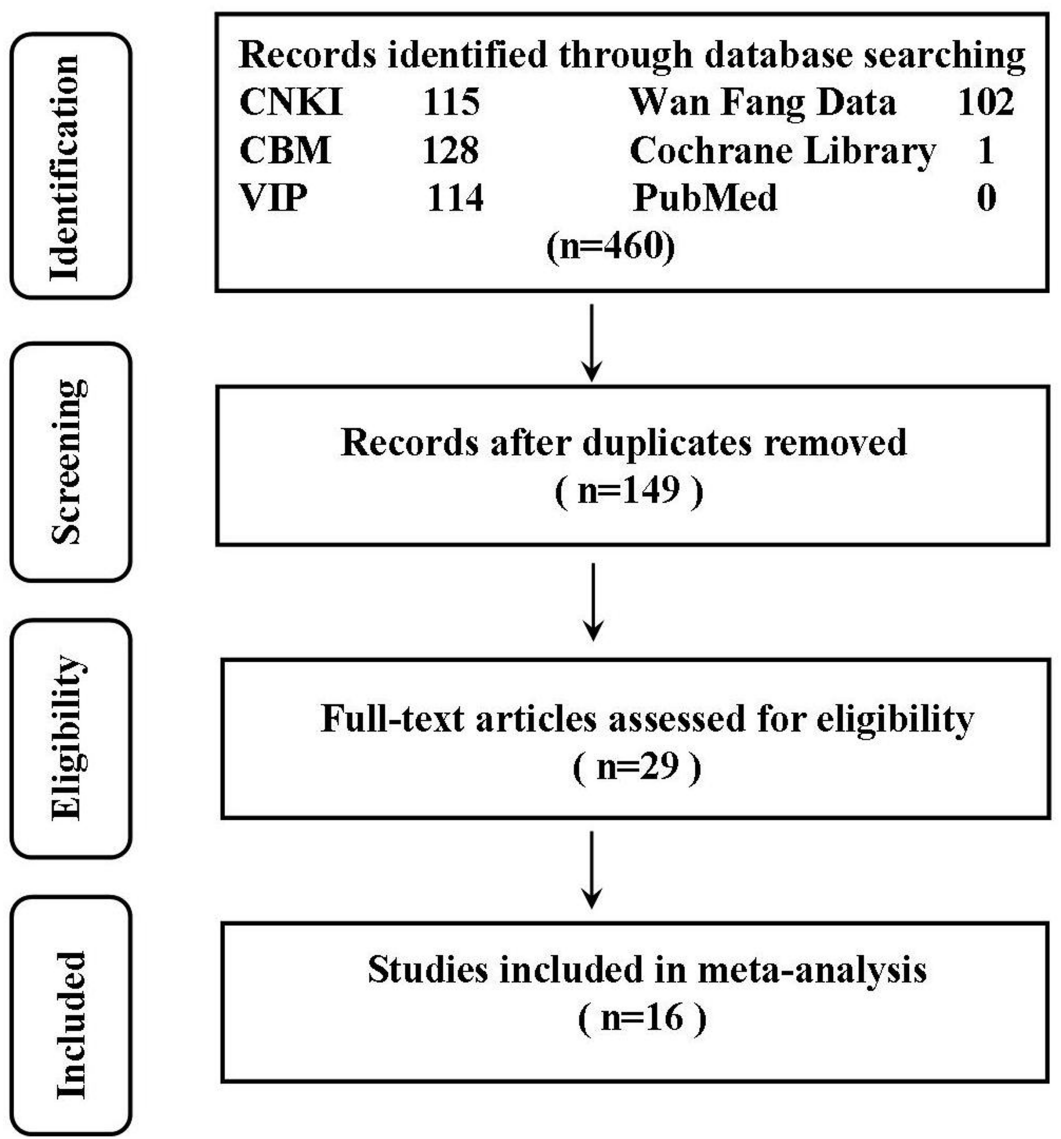
Flow diagram of the reviewed randomized controlled trials.

The process of study selection is shown in Fig 1. The search of biomedical databases yielded 460 studies, wherein 16 matched the inclusion criteria (all in Chinese) [16–31]. There was no disagreement between the two authors regarding the final list of included trials.

### Study Characteristics

The 16 studies were published from 2001 to 2012 and involved 1505 patients with angina pectoris. All documents reported group baseline similarity for the experimental and control groups. The treatment duration lasted for a minimum of 14 days and a maximum of 15 days. The baseline characteristics are shown in Tables 1 and 2.

**Table 1:** Characteristics of the trials included in the meta-analysis.

References	Baseline comparability	Sample size	Outcome Measure	Duration
Deng K 2010	Reported	40	SYM, ECG, hs- *CRP*	14d
Hu S 2011	Reported	100	SYM, ECG	14d
Li HP 2011	Reported	102	SYM, ECG	14d
Liang TH 2010	Reported	220	SYM, ECG	15d
Lu YJ 2010	Reported	80	SYM, ECG	14d
Meng LC 2012	Reported	80	SYM, ECG, hs-CRP, IL-6	14d
Peng DY 2011	Reported	62	SYM, ECG, hemorheology	14d
Shao ZB 2009	Reported	69	SYM, ECG	14d
Shi SH 2011	Reported	82	SYM, ECG	14d
Tan ZH 2009	Reported	149	SYM, ECG	14d
Xu XR 2006	Reported	80	SYM, ECG	14d
Yu HW 2001	Reported	68	SYM, ECG, BP, HR, hemorheology	15d
Zhang QM 2012	Reported	50	SYM, ECG, HL, hemorheology	14d
Zhang XD 2011	Reported	143	SYM, ECG	14d
Zhao JG 2010	Reported	60	SYM, ECG	14d
Zhou BX 2007	Reported	120	SYM, ECG	14d

SYM: symptoms; ECG: electrocardiography; hs-CRP: high-sensitivity C-reactive protein; BP: blood pressure; HR: heart rate.

**Table 2:** Main characteristics of the included studies.

Study	Intervention	
	Control	Treatment
Deng K 2010	Aspirin 100 mg/d, Low molecular heparin sodium 4000 IU/d, Metoprolol 25 mg/d, Simvastatin 20 mg/d, Nitroglycerin 0.3–0.6 mg /d.	Aspirin 100 mg/d, Low molecular heparin sodium 4000 IU/d, Metoprolol 25 mg/d, Simvastatin 20 mg/d, Nitroglycerin 0.3–0.6 mg/d; Breviscapine injection (50 mg/d)
Hu S 2011	Calcium channel blocker, β-blocker, Oxygen Inhalation	Calcium channel blocker, β-blocker, Oxygen Inhalation; Breviscapine injection (50 mg/d)
Li HP 2011	Isosorbide Mononitrate 40 mg/d, Aspirin 75 mg/d, Metoprolol 25 mg/d, Simvastatin 20 mg/d	Isosorbide Mononitrate 40 mg/d, Aspirin 75 mg/d, Metoprolol 25 mg/d, Simvastatin 20 mg/d; Breviscapine injection (40 mg/d)
Liang TH 2010	Nitroglycerin 10 mg/d, Aspirin 75 mg/d	Nitroglycerin 10 mg/d, Aspirin 75 mg/d; Breviscapine injection (30 mg/d)
Lu YJ 2010	Nitroglycerin, Aspirin, β-blocker, ACEI	Nitroglycerin, Aspirin, β-blocker, ACEI; Breviscapine injection (50 mg/d)
Meng LC 2012	Aspirin 100 mg/d, Low molecular heparin sodium 5000 IU/d, Metoprolol 50 mg/d, Atorvastatin 20 mg/d	Aspirin 100 mg/d, Low molecular heparin sodium 5000 IU/d, Metoprolol 50 mg/d, Atorvastatin 20 mg/d; Breviscapine injection (40 mg/d)
Peng DY 2011	Isosorbide dinitrate 5–20 mg/d, Lipid-lowering, antihypertensive, hypoglycemic treatment	Isosorbide dinitrate 5–20 mg/d, Lipid-lowering, antihypertensive, hypoglycemic treatment; Breviscapine injection (50 mg/d)
Shao ZB 2009	Aspirin, nitrates, Low molecular heparin sodium, Statin	Aspirin, nitrates, Low molecular heparin sodium, Statin; Breviscapine injection (100 mg/d)
Shi SH 2011	Aspirin, Calcium channel blocker, β-blocker, ACEI, Nitrates	Aspirin, Calcium channel blocker, β-blocker, ACEI, Nitrates; Breviscapine injection (50 mg/d)
Tan ZH 2009	Nitroglycerin 5 mg/d, Calcium channel blocker, β-blocker	Nitroglycerin 5 mg/d, Calcium channel blocker, β-blocker; Breviscapine injection (25 mg/d)
Xu XR 2006	β-blocker, Dual anti-platelet therapy, vasodilator therapy	β-blocker, Dual anti-platelet therapy, vasodilator therapy; Breviscapine injection (20 mg/d)
Yu HW 2001	Aspirin, Nitroglycerin, Isosorbide dinitrate	Aspirin, Nitroglycerin, Isosorbide dinitrate; Breviscapine injection (100 mg/d)
Zhang QM 2012	Aspirin, β-blocker, ACEI, Nitrates, Lipid drugs	Aspirin, β-blocker, ACEI, Nitrates, Lipid drugs; Breviscapine injection (40 mg/d)
Zhang XD 2011	Aspirin, Nitrates, Low molecular heparin sodium, Statin	Aspirin, Nitrates, Low molecular heparin sodium, Statin; Breviscapine injection (30 mg/d)
Zhao JG 2010	Aspirin 100 mg/d, Low molecular heparin sodium	Aspirin 100 mg/d, Low molecular heparin sodium; Breviscapine injection (100 mg/d)
Zhou BX 2007	Aspirin, nitrates, Dual anti-platelet therapy	Aspirin, nitrates, Dual anti-platelet therapy; Breviscapine injection (50 mg/d)

### Quality Evaluation

According to the selection criteria from the Jadad measuring scale, the 16 studies all scored 2 points and were referred to as random, but lacked random methods. None of the included studies reported whether blind trials were performed. All patients finished the two week treatment.

### Comparison of breviscapine plus Western medicine and Western medicine alone

**Fig 2:**
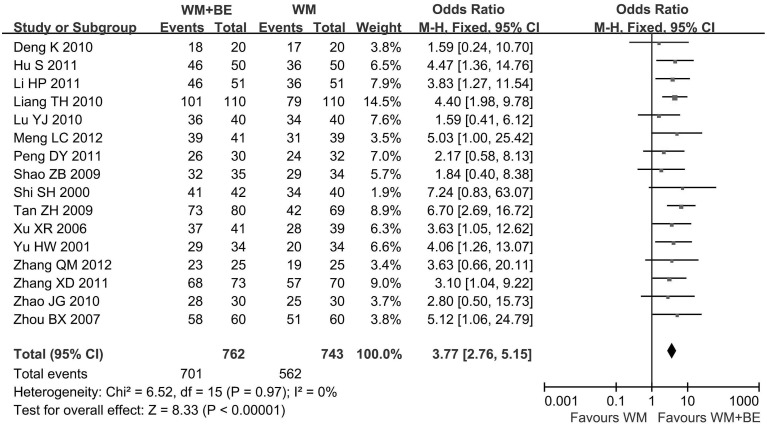
The improvement rate of angina pectoris symptoms: comparison of breviscapine plus Western medicine with Western medicine alone. WM, Western medicine; BE, breviscapine; CI, confidence interval; Test for heterogeneity: chi-squared test with degrees of freedom (d.f.) and P-value; Inconsistency among results: I^2^ test for overall effect; Z statistic with P-value.

**Fig 3:**
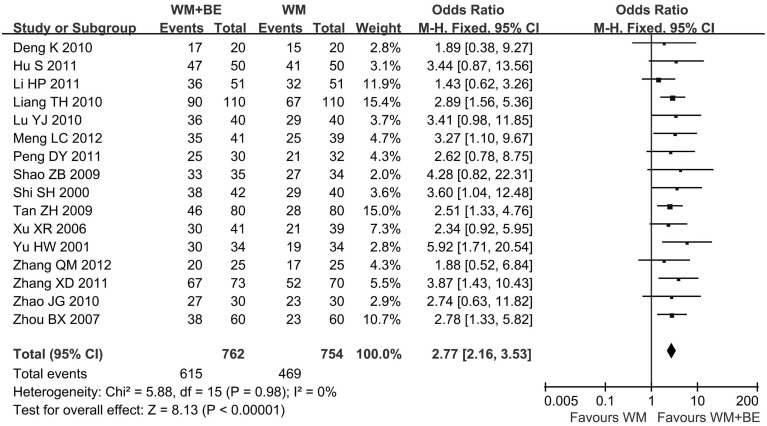
The electrocardiogram improvement rate: comparison of breviscapine plus Western medicine with Western medicine alone. WM, Western medicine; BE, breviscapine; CI, confidence interval; Test for heterogeneity: chi-squared test with degrees of freedom (d.f.) and P-value; Inconsistency among results: I^2^ test for overall effect; Z statistic with P-value.

In this meta-analysis, the combined therapies of breviscapine and Western medicine were superior to Western medicine alone. Overall, the improvement rate of angina pectoris symptoms was higher for patients treated with breviscapine plus Western medicine compared to patients treated with Western medicine alone (92% vs. 76%, respectively, OR, 3.77; 95% CI: 2.76–5.15; p<0.05) (Fig 2). The addition of breviscapine to Western medicine resulted in a 16% increase in the improvement rate compared to patients who underwent Western medicine alone. Combined therapy was also superior to the Western medicine monotherapy in achieving electrocardiogram improvement. The addition of breviscapine led to a 19% increase in ECG improvement compared to patients who underwent Western medicine alone (81% vs. 62%, respectively, OR, 2.77; 95% CI: 2.16–3.53; p<0.05) (Fig 3). The heterogeneity was not significant between the included trials for this meta-analysis.

### Safety evaluation

There was no patient stop the treatment due to adverse effects. Some adverse events were reported in the included trials (including headache, erubescence, Itching skin, palpitations, and fatigue). In the included trials, overall adverse events occurred less frequently for combined therapy than for Western medicine alone.

### Publication bias

**Fig 4:**
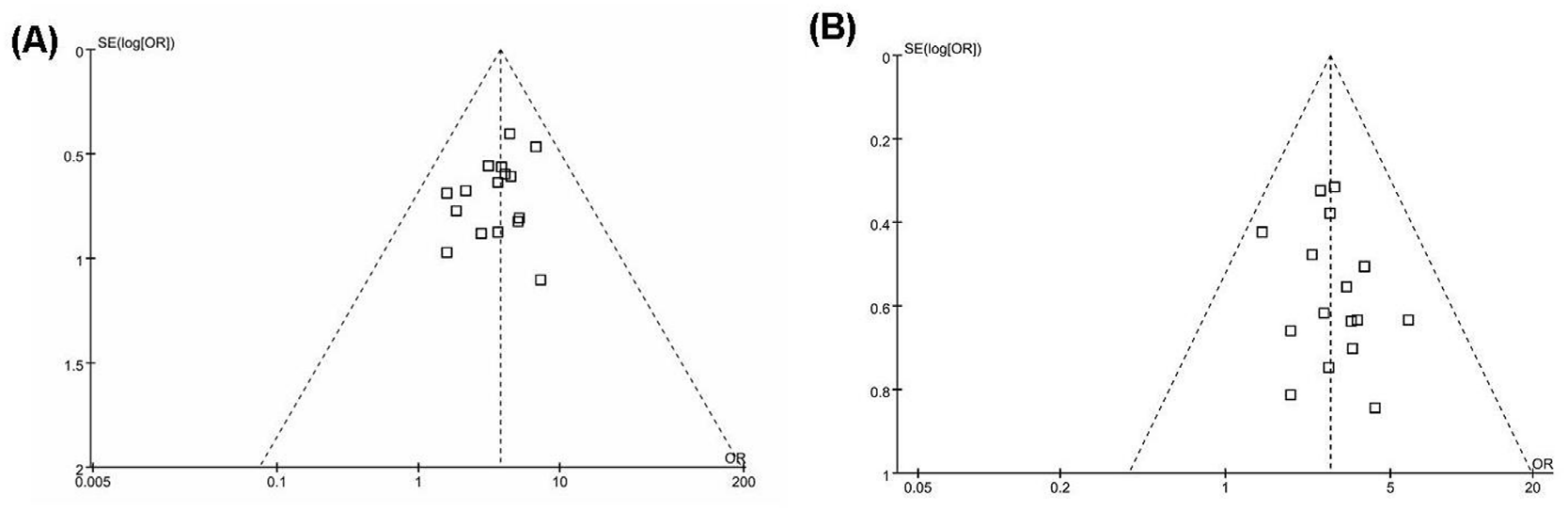
Funnel plot illustrating the included trials used in this meta-analysis. The dashed lines represent the 95% confidence intervals. (A) The improvement rate of angina pectoris symptoms in the included studies. (B) The electrocardiogram improvement rate in the included studies.

Fig 4 shows a funnel plot of the included trials used in the meta-analysis. A funnel plot is a scatter plot of the treatment effects estimated from the individual studies plotted on the horizontal axis against the standard error of the estimate shown on the vertical axis. All trials included in the meta-analysis lie within the 95% CI line, implying the existence of acceptable publication bias.

## Discussion

In Asia, and especially in China, complementary and alternative medicine (CAM) has been introduced to increase the virological response of patients and improve the adverse effects of treatments [13]. As one of the main components of CAM, Chinese medicine has been used as a front line medicine and widely utilized in medical systems. The widespread use of CAM is emphasized among people with chronic diseases [32]. Despite different available therapies, there are still many unmet needs in the management of angina patients. It has become a serious public health problem worldwide, both in developed and developing countries. In most areas of China, it is common to treat angina pectoris patients with a combination of Western medicine and Chinese herbal medicine. With the modernization of Chinese traditional medicine and technology, an increasing amount of Chinese herbal medicine is used clinically, especially at primary care hospitals.

Breviscapine is a common Chinese herb extract used for the treatment of angina pectoris and is widely used in China. Experimental studies have shown that Breviscapine can eliminate free radicals and display antioxidant, antiplatelet aggregation, anti-ischemic, and anti-arrhythmic effects and inhibit the proliferation of vascular smooth muscle [9–12]. The results of the current meta-analysis show that patients benefited from the addition of Breviscapine injections to Western medicine therapy. No obvious adverse reactions have been observed, and the combination therapy is safe, reliable and superior to conventional Western medicine alone. Although all of the selected studies claimed that combination therapy can improve blood lipid measurements, the meta-analysis showed no statistical significance (data not shown). Therefore, there is no evidence that combination therapy has advantages in improving blood lipid values. Large sample of RCTs are needed to provide further evidence. In this study, the results showed that the combined therapy of Breviscapine plus Western medicine was superior to Western medicine alone for improving both angina pectoris symptoms and ECG findings. Rare severe side effects have been reported for Breviscapine (including headache, erubescence, Itching skin, palpitations, and fatigue), but the current evidence shows that Breviscapine is quite safe [17, 20, 22]. The current evidence suggests that Breviscapine plus Western medicine achieved superior therapeutic effects compared to Western medicine alone, and Breviscapine does not result in any additional safety problems.

It is should be mentioned that there were some limitations in this study. There is insufficient data to perform a sub-group analysis. The quality of the included RCTs in this meta-analysis was not high because the full accounts of all randomized patients, follow-up evaluations and blinded methods were not described. Although the main worldwide biomedical databases were searched to identify potential RCTs, publication bias could not be completely avoided. The results of this meta-analysis showed no apparent heterogeneity, and the direction of the treatment effect is the same across all included trials. Due to the revise of western treatment therapies in recent years, some included studies are out of date. This might also contributed the bias in this study.

## Conclusion

The current evidence suggests that Breviscapine plus Western medicine achieved superior therapeutic effects compared to Western medicine alone.

## Supporting Information

S1 PRISMA flow chart. (DOC)

S2 PRISMA Checklist. (DOC)
