# Supplementary material for: Breviscapine Injection Improves the Therapeutic Effect of Western Medicine on Angina Pectoris Patients
Source: PLoS One. 2015 Jun 8;10(6):e0129969. doi: 10.1371/journal.pone.0129969 (PMC4460136; doi:10.1371/journal.pone.0129969)
Supplement: S1 PRISMA — (DOC) [file pone.0129969.s001.doc]

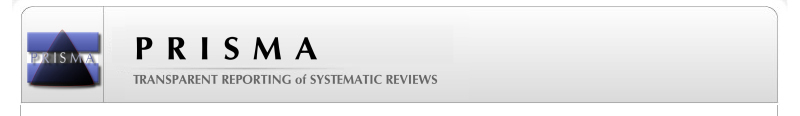
**PRISMA 2009 Flow Diagram**

**Screening**

**Included**

**Eligibility**

**Identification**

Records identified through database searching
(n =616 )

Additional records identified through other sources
(n = 0 )

Records after duplicates removed
(n =149 )

Records screened
(n = 96 )

Records excluded
(n =57 )

Full-text articles assessed for eligibility
(n =29 )

Full-text articles excluded, with reasons
(n = 13 )

Studies included in qualitative synthesis
(n = 16 )

Studies included in quantitative synthesis (meta-analysis)
(n =16 )
